# Partnerships Matter in Successful Aging: Collaborations That Put Research Into Practice Across the Community

**DOI:** 10.1093/geroni/igaa057.2850

**Published:** 2020-12-16

**Authors:** Tina Sadarangani, Bei Wu

## Abstract

Healthy aging begins and ends in the community with age friendly health systems and communities. In order to promote healthy aging and help older adults make healthy choices we must engage them as partners in healthcare with access to appropriate information and resources. This symposium will examine four community based studies that engage stakeholders to improve care quality through evidence-based interventions for older adults. These programs are all novel as they seek to engage stakeholders, perform pragmatic interventions, and improve outcomes in non-academic, community-based settings which are often overlooked. Promoting healthy behavior through peer coaching, using early detection and treatment to impact cognitive decline in social day care programs, evaluating the feasibility of screening for palliative care in assisted living and implementing evidence-based dementia care in hospice settings are all explored. Analysis of these initiatives showed improvements in perceived-health, reductions in unnecessary healthcare utilization, and improvements in the physical and emotional health of caregivers, and positive changes in health behaviors. Our discussion will underscore the importance of engaging key stakeholders in study design and implementation to yield better outcomes. Community engagement is an essential part of facilitating aging-in-place, and findings illustrate that this can be achieved through innovative collaborations between researchers and community-based organizations across a variety of settings.

